# Editorial: Use of Botox in Abdominal Wall Hernia Surgery

**DOI:** 10.3389/jaws.2026.16597

**Published:** 2026-04-09

**Authors:** Pilar Hernández Granados, Mette Willaume Christoffersen

**Affiliations:** 1 General and Digestive Surgery Department, Hospital Universitario Fundación Alcorcón, Alcorcón, Spain; 2 Sjaellands Universitetshospital Koge, Køge, Denmark

**Keywords:** abdominal wall surgery, botulinum toxin A (BTA), prehabilitation, progressive pneumoperitoneum, secondary effects of BTA

Since the first publication in 2009 describing the use of Botulinum Toxin A (BTA) in abdominal wall reconstruction, its use as an adjunct to the prehabilitation strategy for patients with abdominal wall hernias has increased worldwide. Evidence suggests that BTA may help closing the fascial defects and thereby avoid bridging with mesh, and may even reduce the need for component separation techniques (CST), which are associated with significant morbidity, and most important, diminish the risk of abdominal compartment syndrome. Furthermore, the adverse effects of BTA appear to be minimal and uncommon.

However, after more than 15 years of widespread use, the optimal dose, the number of injections per side of the abdominal wall, the appropriate muscular plane for injection and many other important questions remain to be answered. In addition, indications for BTA as a prehabilitation tool vary across different clinical settings. The most common indication is midline hernias with wide defects (≥10 cm, W3 according to the EHS classification) and/or in patients with loss of domain. Favourable outcomes in this patient population have encouraged its use in smaller transverse defects, circular defects that are traditionally difficult to close, hernias out of the midline, giant inguinoscrotal hernias, open abdomen management, and other complex scenarios.

This Special Issue addresses and describes the multiple aspects of BTA use in abdominal wall reconstruction. BTA is frequently combined with other prehabilitation strategies, such as preoperative progressive pneumoperitoneum (PPP) and intraoperative fascial traction (IFT) and most specialized centers managing complex hernias have developed structured algorithms for patient management.

Marques-Antunes et al. propose a preoperative planning strategy based on CT findings for complex midline hernias. BTA was indicated 4–6 weeks prior to surgery when the defect width was ≥8 cm, lateral muscle thickness ≥1 cm, or loss of domain (LOD) was present (Tanaka index >0.20). PPP was added in cases with LOD. In W3 hernias with significant lateral muscle retraction, IFT was recommended. Using this algorithm, CST was avoided in 61.4% of patients.

Taking this approach one step further, Nip et al. describe the establishment of a dedicated BTA service in a tertiary hernia center in the United Kingdom. A multidisciplinary team developed an algorithm recommending BTA injection for transverse defects measuring 5–10 cm when associated with additional risk factors, and for all defects >10 cm. Only 51% of patients required CST, and fascial closure was achieved in 91% of cases.

Conversely, Girieasen et al. applied a different algorithm, reserving BTA (combined with PPP) for cases with a Tanaka index >0.35. All patients managed under this protocol ultimately required CST, possibly reflecting its use exclusively in highly complex cases.

Bustamante-Recuenco et al. describe the use of BTA combined with a novel IFT device in a patient with a large 8-cm umbilical hernia associated with rectus diastasis. A preperitoneal enhanced-view totally extraperitoneal (Pe-TEP) approach was performed using this new traction system, achieving successful defect closure.

Sánchez-Moreno et al. report an unusual indication for BTA: three cases of severe diaphragmatic paralysis in which a substantial volume of abdominal contents had migrated into the left hemithorax, resulting in significant respiratory impairment. Four weeks after BTA injection, patients underwent diaphragmatic plication with mesh reinforcement. This strategy prevented the development of postoperative abdominal compartment syndrome. Recovery was uneventful, and all patients experienced improved quality of life.

Most publications report few complications related to BTA administration, aside from occasional injection-site hematomas. While the anatomical and muscular benefits of BTA as a prehabilitation tool are well documented, its potential effects on pulmonary function remain insufficiently studied, despite the role of lateral abdominal muscles in respiration. Smietanski et al. evaluated the impact of BTA on respiratory function using spirometry in 37 patients to determine whether observed changes represented true physiological improvement, mechanical compensation, or possible impairment. The authors conclude that BTA appears safe from a respiratory standpoint, as core spirometric parameters were not adversely affected. However, conventional spirometry may not fully capture BTA-induced functional changes, highlighting the need for further investigation.

There is, however, limited information regarding patients’ perspectives and experiences with BTA. Klein et al. report the results of a questionnaire administered to 22 patients with complex hernias treated with preoperative BTA prior to abdominal wall reconstruction. Injection-related pain was infrequent; three patients reported no pain at all. Eight patients noticed changes in abdominal contour, two described altered trunk function, one experienced mild dyspnea, and another reported constipation. Overall, 59% of patients described positive memories and good tolerance of the procedure. These findings are consistent with the first international survey on patient-reported outcomes following preoperative BTA administration, recently published.

Although this Special Issue does not address all unresolved questions regarding BTA use in abdominal wall reconstruction, it provides a comprehensive overview of its global application, primarily within structured management algorithms for complex hernias. Patient-reported outcomes further support the notion that BTA is generally well tolerated, with few adverse effects. Nevertheless, many questions remain unanswered.

We hope this special issue will be both engaging and informative, contributing to a deeper understanding of the diverse applications and effects within this field, while also encouraging further research and scientific publications in the future.

